# Machine Learning for Early Prediction of Sepsis in Intensive Care Unit (ICU) Patients

**DOI:** 10.3390/medicina59071276

**Published:** 2023-07-09

**Authors:** Abdullah Alanazi, Lujain Aldakhil, Mohammed Aldhoayan, Bakheet Aldosari

**Affiliations:** 1 Department of Health Informatics, College of Public Health and Health Informatics, King Saud Ibn Abdulaziz University for Health Sciences, Riyadh 11481, Saudi Arabia; 2 King Abdullah International Medical Research Center, Riyadh 14611, Saudi Arabia

**Keywords:** sepsis, intensive care unit, machine learning, data mining algorithms, prediction, models, survival analysis, confusion matrixes, performance

## Abstract

*Background and Objectives:* Early detection of sepsis is crucial and can save lives. However, identifying sepsis early and accurately remains a difficult task in the medical field. This study aims to investigate a new machine-learning approach. By analyzing the clinical laboratory results and vital signs of adult patients in the ICU, this approach can predict and detect the initial signs of sepsis. *Materials and Methods:* To examine survival rates and predict outcomes, the study utilized several models, including the proportional hazards model and data mining algorithms. We analyzed data from the BESTCare database at KAMC, with a focus on patients aged 14 and older who were admitted to the ICU between April and October 2018. We conducted a thorough analysis of the medical records of a total of 1182 patients who were diagnosed with sepsis. *Results:* We studied two approaches to predict sepsis in ICU patients. The regression model utilizing survival analysis showed moderate predictive ability, emphasizing the importance of only three factors—time (from sepsis to an outcome; discharge or death), lactic acid, and temperature—had a significant *p*-value (*p* = 0.000568, *p* = 0.01, *p* = 0.02, respectively). Other data mining algorithms may have limitations due to their assumptions of variable independence and linear classification nature. *Conclusions:* To achieve progress and accuracy in the field of sepsis prediction, it is important to continuously strive for improvement. By meticulously cleaning and selecting data attributes, we can create a strong foundation for future advancements in this area.

## 1. Introduction

Sepsis is a widespread medical condition that poses a significant risk to people’s lives worldwide. In 2017, it affected 49 million people and has been linked to 11 million preventable deaths globally [1]. Sepsis is an acute reaction to an infection, often bacterial, which causes physical changes in the patient’s vital signs. These changes may include fluctuations in body temperature, changes in white blood cell count, rapid heartbeat, and difficulty breathing, and can lead to organ failure and death [2]. The progression of sepsis is classified into three distinct stages, each with varying severity and associated risks. The first stage is sepsis, characterized by a mild response to the infection and a relatively low mortality risk. The next stage is severe sepsis, in which the patient’s condition worsens, and the risk of mortality increases significantly. Finally, the most severe and life-threatening stage of sepsis is septic shock, in which the body’s response to the infection becomes overwhelming, leading to a range of complications and multi-organ failure [3].

It is of utmost importance to accurately identify the different stages of sepsis to determine the most effective treatment and management plan for patients. Failure to do so promptly can lead to severe complications and, in some cases, even death. Thus, educating healthcare providers and the public on the signs and symptoms of sepsis is crucial to ensure early detection and intervention. A multicenter cohort study in Colombia revealed intraabdominal and respiratory infections significantly increase the risk of clinical progression of sepsis, underscoring the need for heightened awareness and vigilance in monitoring these infections [3].

Timely identification of patients who may be at risk of developing sepsis is of paramount importance. A delayed diagnosis can lead to a more severe condition, higher mortality rates, and an increased likelihood of developing sepsis. Early interventions can help prevent such outcomes and improve the patient’s overall prognosis [4]. Vigilance for indications of sepsis is essential. Identifying the symptoms early is crucial to promptly take necessary measures and prevent the condition from deteriorating [4]. That approximately 750,000 hospitalized patients in the United States are diagnosed with severe sepsis yearly is alarming [5].

Early diagnosis and treatment have been shown to reduce mortality. Early and accurate sepsis detection is still a challenging clinical problem. When it comes to monitoring patients with sepsis electronically, only a handful of methods exist that can predict and detect early signs of complications such as multi-organ failure [6]. However, one technique that has been developed to address this issue is the implementation of a machine-learning algorithm (MLA) [7]. This approach utilizes machine learning (ML), which allows computers to learn from data without requiring explicit programming [8]. ML can identify patterns and relationships within vast quantities of data, making it a handy tool for solving complex problems related to pattern recognition and data analytics. The origins of this concept can be traced back to Arthur Samuel in 1959 [8].

Data mining is a widely used method in the medical field to classify diseases based on health associations [9]. Machine learning is a recognized tool that aids in predicting outcomes by teaching computers how to learn from data. This technology has transformed data analysis by merging statistical and efficient computing methods. Two main types of computer learning systems are supervised and unsupervised, which have greatly improved the decision-making process [10]. Data analytics incorporates numerous data mining techniques, such as association, classification, clustering, prediction, sequential patterns, and decision tree [11]. A classification technique is utilized in machine learning to predict output based on a pre-determined set of classes or groups. For instance, in the medical setting, patient information is used as the training set, and the predicting attributes are whether the patient will have a disease. Machine learning has garnered significant interest in the medical field because of its ability to detect illnesses rapidly and with less patient interaction, thus saving time in patient care [10].

Several studies have proven the beneficial impact of utilizing machine learning algorithms and predictive models in the context of sepsis mortality rates and the length of hospital stays. A clinical trial conducted on a randomized basis yielded stunning results, indicating that a machine learning algorithm could predict sepsis, reduce the length of stay from 13 days to 10.3 days and decrease mortality by 12.4% [7]. The algorithm employed six vital signs and demonstrated higher sensitivity and specificity compared to other sepsis scoring systems, ultimately contributing to improved patient outcomes and health quality [7]. Furthermore, a separate study at Cabell Huntington Hospital revealed that implementing a machine learning algorithm for early sepsis detection decreased sepsis-related length of stay from 2.99 days to 2.48 days and reduced in-hospital mortality from 3.97% to 2.64%. These findings are indicative of a promising approach toward enhancing patient outcomes [12]. In a recent study, machine learning algorithms proved beneficial in predicting sepsis in ICU patients. The algorithms identified new predictors of sepsis by analyzing clinical laboratory values and vital signs in adult ICU patients. This led to improved outcomes, such as reduced mortality rates and shorter hospital stays. These findings suggest that utilizing machine learning technology can significantly impact the healthcare industry and ultimately improve patient care [13]. A study by Nemati et al. showed how using real-time data from an ICU can help artificial intelligence sepsis experts predict the onset of sepsis in a patient up to 12 h before it is clinically recognized. This is a significant breakthrough in healthcare because it can save lives by allowing doctors to intervene earlier [14]. However, it is essential to conduct further studies to determine the feasibility of implementing this sepsis prediction model in real-world scenarios.

## 2. Materials and Methods

In this study, we employed machine-learning techniques to analyze a dataset and create a prediction model. The research is retrospective, meaning we have focused on using historical data on patients’ vital signs and lab tests. We have meticulously followed the various stages of the data mining process, starting with data collection and preparation, then mining, building the model, and finally testing and validating the model.

During Phase 1 of the study, which took place from April to October 2018, we used databases from a tertiary hospital to collect relevant data. Sepsis cases are identified by a confirmed diagnosis of a life-threatening infection associated with organ dysfunction in the patient’s chart. Suspect cases with a score of 2 or more on SOFA or qSOFA also suggest sepsis. Additionally, cases requiring vasopressors to maintain arterial pressure at 65 mm or greater and having serum lactate more than two mmol/L (with no hypovolemia) are in septic shock. We included in the study patients who met the criteria during this period. In Phase 2, we preprocessed the collected data to resolve any incorrect, incomplete, missing, or inconsistent data issues. This step was crucial in ensuring the data was reliable and accurate for the subsequent analysis. In the third phase of the process, we used techniques such as the proportional hazards regression method and data mining algorithms like logistic, SMO, naïve Bayes, rules (JRip), and k-nearest neighbors (KNN) to create a prediction model. Once we built the model, we subjected it to rigorous testing and validation in Phase 4, using a cross-validation technique. This method involves dividing the dataset into two sets: a training set to train the model and a test set to evaluate the model’s performance. The goal of this phase is to ensure the model is accurate and reliable, which is crucial for its success.

### 2.1. Study Setting

We conducted the investigation at King Abdul-Aziz Medical City (KAMC), a notable healthcare facility in Riyadh that commenced operations in May 1983 and currently houses 1501 beds. KAMC has gradually expanded its services and has gained a reputation for delivering exceptional medical care. It operates under the Ministry of National Guard Health Affairs (MNGHA). The research specifically examined adult patients admitted to the Intensive Care Unit, with and without sepsis.

### 2.2. Participant Selection

We aimed to examine a group of ICU patients aged 14 or older, considering demographic information such as age and gender and laboratory results encompassing lactic acid, blood sugar, and WBC. We also used nursing assessments, including measuring SBP, temperature, pulse, respiratory rate, and SpO2. Although an elevated C-reactive protein (CRP) indicates infection, its specificity is questioned for diagnosing sepsis. However, including more biomarker for early prognostication in septic shock patients in future studies are recommended. The study included all ICU patients, with and without sepsis, who had laboratory tests between April and October 2018.

### 2.3. Data Collection

We endeavored to identify the benefits of using machine learning techniques to predict the early onset of sepsis and assess the impact of timely intervention on mortality rate and duration of hospital stay. We sourced the information from the BESTCare database at KAMC. Notably, we assessed 1182 patient records between April and October 2018 before we began data preprocessing. The study focused on ICU patients aged 14 years and older, encompassing 11 data categories. These categories included essential patient information like gender, laboratory records, vital signs, and the sepsis label, denoted by two values (0 or 1).

In healthcare data mining, data preprocessing is integral in ensuring a high quality of collected data. Healthcare organizations gather information from various sources, which often results in inconsistencies, noise, and missing values. To refine and improve the data quality, we subjected the collected dataset to a rigorous preprocessing phase, including assessing and verifying its reliability and validity. We thoroughly checked the dataset and excluded irrelevant and redundant attributes, representing more than 50% of the total number of instances. This has helped to improve the comprehensibility of the prediction model. Our preprocessing phase consisted of several steps: 1. Dimensionality: we removed attributes that had null values and were irrelevant or redundant. 2. attributes with many values: For attributes such as lab tests (WBC, Blood sugar, Lactic acid) and nurse’s assessment (Temperature, RR, HR, Spo2), we calculated the median of each attribute to be the value. 3. missing values: We manually completed missing values by using the median value of the attribute. 4. attribute with dates: The dataset contained three attributes for timing: time to sepsis, time to discharge, and time to death. We calculated the dates in hours and combined them into one column, prioritizing time to sepsis, death, and discharge. The attributes were selected by examining the literature and getting input from intensive care doctors. However, a high C-reactive protein (CRP) level was excluded. It is advisable to include more biomarkers in upcoming research to enhance the early prognosis for sepsis patients.

### 2.4. Data Mining

We tested different prediction models in the data mining phase, such as logistic, SMO, naïve Bayes, JRip, and KNN. We also used proportional hazard regression for survival analysis. We classified individuals as having sepsis (1) or not (0). Survival analysis involves analyzing data in which the outcome variable is the time until a specific event occurs. Proportional hazard regression is the most widely used method for studying how predictor variables impact survival time [11]. Logistic regression classifies observations using a model and probability estimates, which is effective for categorical data [15]. In machine learning, support vector machines (SVM) are models that can tackle classification or regression challenges in a supervised learning setting. Among the types of SVM is sequential minimal optimization (SMO), which is particularly efficient in solving classification problems and handling regression issues [16]. The Naïve Bayes algorithm is a probabilistic classifier for classification problems. It is based on Bayes’ probability theorem. Our study used the Naïve Bayes algorithm to analyze the classifier output for predicting each dataset instance. We used Repeated Incremental Pruning to Produce Error Reduction (RIPPER) and the (JRip) algorithm to analyze, classify, and generate rules for each class individually, then repeated the process for all subsequent classes [17]. KNN is a simple algorithm for regression and classification in machine learning. It divides the dataset into classes for prediction [18]. The attributes were selected by examining the literature and getting input from intensive care doctors. Although a high C-reactive protein (CRP) level can indicate an infection, it may not be precise enough to identify sepsis. It is advisable to include more biomarkers in upcoming research to enhance the early prognosis for sepsis patients. The study includes ten attributes: gender, time, blood sugar, lactic acid, SBP, SpO2, heart rate, white blood cell count, temperature, and respiratory rate. We conducted the experiments using split data and internal cross-validation with ten folds.

## 3. Results

We conducted a thorough analysis of the medical records of a total of 1182 patients who were diagnosed with sepsis. To ensure the validity of our findings, we only included patients who were 14 years or older and were admitted to the intensive care unit (ICU) at the KAMC. In this study, we explored two approaches for predicting sepsis in its early stages. The first technique uses a regression model known as survival analysis, and the second uses a data mining algorithm. Our dataset comprises valuable information on vital signs, lab tests, and demographic characteristics. However, the dataset is primarily dominated by negative instances, with only a few positive ones. This class imbalance poses a significant challenge for data mining because most algorithms focus on classifying large samples and disregarding minority samples. Nonetheless, in some cases, the minority samples are critical for accurate predictions. To overcome this issue, we opted to use the under-sampling technique.

In our study, we utilized a regression model called survival analysis to predict the likelihood of sepsis in patients in the ICU during the early stages of their illness. Table 1 presents the results of this analysis, with time used as an outcome variable. We used a confusion matrix to further evaluate the model’s performance, revealing that the model’s ability to predict sepsis could have been more accurate. Our research focuses on the elapsed time between the onset of an event and the development of sepsis. We employ proportional hazard models to determine the factors that contribute to the development of sepsis. In this type of model, the impact of each factor is measured by the extent to which it increases the hazard rate. For instance, a rise in body temperature elevates the hazard rate for sepsis by 75%, while the white blood cell count has no effect. According to our findings, three factors, namely time (from sepsis to an outcome; discharge or death), lactic acid, and temperature, significantly impact sepsis development (*p* = 0.000568, *p* = 0.01, *p* = 0.02, respectively). We re-evaluated the model using these three factors, and the confusion matrix confirmed our initial results.

The parameter estimates show how much the expected log of the relative hazard increases for each unit increases in the predictor while keeping other predictors constant. Specifically, for every degree increase in temperature, there is a 0.200 unit increase in the expected log of the relative hazard while holding lactic acid and time constant. Additionally, for every increase in time (in a day), there is a 0.635 unit increase in the expected log of the relative hazard while holding temperature and lactic acid constant. Finally, for every increase in lactic acid, there is a 0.104 unit increase in the expected log of the relative hazard while holding temperature and time constant. The estimates for all parameters were calculated while considering the other predictors. When temperature, lactic acid, and time (days) were considered, no statistically significant links were found between any of the other attributes (gender, age, blood sugar, WBC, SBP, RR, and SpO2) and all-cause mortality. This does not necessarily mean these risk factors are not linked to all-cause mortality. The lack of significance is probably due to confounding, which refers to the risk factors’ interrelationships.

In the second method, we used data mining algorithms such as logistic, SMO, naïve Bayes, rules (JRip), and k-nearest neighbors (KNN) to predict sepsis patients in this study. We used accuracy, F-measure, recall, precision, and ROC (Receiver Operating Curve) measures to find the best performance for the prediction. The accuracy of the models ranged between 77% and 89%. Logistics showed a maximum accuracy of 89.19%, and Naive bays offered the lowest accuracy rate, 77.27%. The recall, precision, and F-measure of algorithms achieved over 80%. The SMO, IKB, and JRip algorithms showed a ROC percentage of over 50%, while naive Bayes and logistics achieved over 69%, as Table 2 shows.

Table 3 shows that the model predicts almost all patients with no sepsis (negative class). It also shows, however, that the model cannot predict those who have sepsis (positive class), which is the focus of this study. Furthermore, the results show that three attributes have a significant *p*-value: time, temperature, and lactic acid. We rebuilt the model using these attributes, and the confusion matrix showed no difference in the results. The prediction of sepsis patients is still low, as Table 2 shows.

## 4. Discussion

The main aim of this study was to create a model that could effectively predict the onset of sepsis at an early stage. For this purpose, two distinct methods were employed on the available dataset—a survival analysis (in the form of a regression model) to forecast the early onset of sepsis and a data mining algorithm to classify whether the patient would develop sepsis. Prior research has emphasized that the use of machine learning algorithms in the medical field can significantly enhance the quality of care and reduce both mortality rates and the length of hospital stays. Timely detection of sepsis can help medical practitioners initiate appropriate treatments, including antibiotics, fluids, and vasopressors, and allow them to identify the underlying causes of the ailment [7,12,14].

In medical diagnostics, predicting sepsis in its early stages is of utmost importance. Establish an early warning system in which alerts can be fired and further confirmed by clinicians has decreased the mortality rate from 19.2% to 14.6% and reduced length of stay from 8.1 to 6.6 days, with the ability to detect patients at lower SOFA scores (4 in compared to 4.3) in one multicenter study [19]. A regression model utilizing survival analysis helps achieves this. Upon reviewing the relevant attributes related to sepsis, an increased morality rate was associated with age, the severity of sepsis, lactate level, coagulopathy, and other factors [20]. Further, respiratory, circulatory, and other organ dysfunctions are associated with the introduction of vasopressors, ventilation, systolic blood pressure, urine output, and lactate level [21]. With the counseling of clinicians, especially intensivists, a model was built including ten attributes: gender, Time, Blood Sugar, Lactic Acid, SBP, SpO2, HR, WBC, Temperature, and Respiratory rate. The number of attributes in the model is similar to other studies aimed to serve the same purpose, Abromavicius and others in their study have used 11 variables, including demographics, labs, and vital signs data, while Calvert’s study utilized nine variables, and Desautels study used eight variables covering demographics and vital signs data [22,23,24]. Further, the Barton study used only six vital signs attributes (SpO2, heart rate, respiratory rate, temperature, systolic and diastolic blood pressure) and revealed superior predictability in comparison with commonly used approaches [25].

The model’s performance is evaluated using a confusion matrix, as Table 1 shows. Upon examination, it becomes evident that the model effectively identifies patients who do not have sepsis (negative class) but struggles to predict those who do (positive class). These findings are similar to the findings of the study of Lin PC and others, as their model diagnostic performance of sepsis has high NPV (ranges between 0.77 to 0.84) and low PPV (0.34 to 0.76 depending on the used algorithms) [26]. It should be noted that the study focuses specifically on early prediction of sepsis. In sepsis prediction, the conventional approach has been to rely on extensive databases. However, every sepsis case is unique, resulting in a significant amount of variability among patients. One of the main challenges of sepsis detection lies in the differentiation between confirmed sepsis cases and non-sepsis patients. This classification process can be arduous in clinical settings because of the differences in patients’ symptoms and treatments. Recognizing that training a particular dataset with information from all patients might be misleading given these discrepancies is crucial [27].

The study’s results highlight three significant attributes crucial in predicting the onset of sepsis: time, temperature, and lactic acid. All three of these attributes possess a significant *p*-value. Considering this, we restructured the model using these attributes. However, as Table 2 notes, there is still room for improvement in predicting sepsis patients.

This study used various data mining algorithms to predict sepsis patients, including logistic, SMO, naïve Bayes, rules (JRip), and k-nearest neighbors (KNN). Table 1 displays each algorithm’s Accuracy, F-Measure, Recall, Precision, and ROC (Receiver Operating Curve) measures. We used these measures to evaluate the performance of each algorithm. However, despite efforts such as preprocessing, balancing data, and utilizing significant attributes from the first model, the confusion matrixes for these algorithms did not accurately predict sepsis. Our findings indicate a moderate prediction rate for sepsis patients. Compared to traditional predictive tools used in single studies, machine learning models had a higher score in AUROC, mostly scoring above 0.6 and even reaching 0.9 in some cases [25,28]. This is significantly comparable to our predictive tools, which scored about 0.7. Additionally, two studies detected sepsis using predictive models with an AUROC value of about 0.9 [29,30]. This shows a solid ability to differentiate between sepsis and non-sepsis patients within zero hours, which indicates when the last sign of severe sepsis is noted, showing a potential infection, all within six hours.

Based on these results, we can confidently say that machine learning algorithms effectively predict sepsis. Nevertheless, clinical researchers can gather essential data daily to study the dynamic progression of sepsis effectively. When combined with their clinical expertise, this data can offer valuable insight into the disease’s evolution. Furthermore, a machine learning model powered by neural networks can be employed to simulate the development of sepsis. This model can predict a patient’s prognosis, which can be extremely helpful in determining the best course of treatment [31].

This study’s dataset posed a challenge and had limitations. High-quality data is pivotal for machine learning, and any inconsistency, inaccuracy, or missing data can significantly impact algorithm performance. In this regard, preprocessing the dataset is the first and most crucial step before implementing the algorithms. Moreover, it is worth noting that the study focused on only one hospital, KAMC, and a specific department (ICU); consequently, we retrieved only a few records from the database. This limited dataset may yield low-performance results, and more attributes are necessary to improve predictions of sepsis in its early stages. Monitoring patients and taking their measures every hour can be highly beneficial in further bolstering the model’s performance. To improve future studies on sepsis cases, it is recommended to involve expert clinicians in the data preprocessing phase due to the unique nature of each case and the challenges of consolidating patient data into one dataset. To improve the generalizability of research findings, using data sets compiled from multiple hospitals or centers with standardized data quality would be beneficial. Additionally, assessing the practicality of incorporating machine learning models into clinical workflow and measuring the resulting patient outcomes would be helpful.

## 5. Conclusions

Early detection of sepsis is crucial in healthcare, and recent research has explored new ways to predict its onset accurately. Researchers have analyzed data from one tertiary hospital database using advanced machine learning algorithms and survival analysis. Based on our research, machine learning models are better at predicting sepsis in patients than traditional methods. However, the model is more reliable at identifying patients who do not have sepsis than those who do, which is consistent with other studies. Our study emphasizes that sepsis can be anticipated by three crucial factors: time, temperature, and lactic acid levels. Although the results were moderate, efforts are underway to improve the models by applying more data preprocessing and attribute selection. The study will expand to settings beyond intensive care units and extend the analysis period. These efforts will help enhance future models and improve early detection of sepsis, ultimately saving more lives.

## Figures and Tables

**Table 1:** Proportional hazards mode.

Attributes	Parameter Estimate	*p*-Value	Hazard Ratio (HR)
Gender	0.625	0.73	0.90
Age (years)	0.327	0.62	0.99
Blood Sugar (Mdn)	0.296	0.59	0.96
Lactic Acid (Mdn)	0.104	0.01	1.16
WBC (Mdn)	0.221	0.55	0.96
Spontaneous bacterial peritonitis (SBP) (Mdn)	0.424	0.73	0.99
Temperature (Mdn)	0.200	0.02	1.75
Respiratory Rate (Mdn)	0.621	0.68	1.03
Oxygen saturation (SpO2) (Mdn)	0.709	0.71	1.01
Time (days)	0.635	0.001	1.01

**Table 2:** Comparative Performance of Classification Algorithms on Different Measures.

Algorithms	Accuracy	Precision	Recall	F-Measure	ROC
Naïve Buys	77.27	0.850	0.773	0.803	0.690
Logistic	89.19	0.863	0.892	0.860	0.750
SMO	88.88	0.889	0.889	0.941	0.500
IKB	83.33	0.829	0.833	0.831	0.564
JRip	88.78	0.861	0.888	0.867	0.572

**Table 3:** Confusion Matrix of regression models.

	No-Sepsis	Sepsis
Regression Model 1		
Tested Negative	803	32
Tested Positive	0	1
Regression Model 2		
Tested Negative	802	31
Tested Positive	0	2

## Data Availability

Not applicable.
